# Distrusted disclosures: Deception drives anti-transgender but not anti-atheist prejudice

**DOI:** 10.3389/fpsyg.2022.1006107

**Published:** 2023-01-26

**Authors:** Rebecca R. Totton, Kimberly Rios, Nathaniel Shogren

**Affiliations:** ^1^Department of Psychology, Amherst College, Amherst, MA, United States; ^2^Department of Psychology, Ohio University, Athens, OH, United States

**Keywords:** deception, distrust, transgender, atheist, prejudice

## Abstract

**Introduction:**

Transgender individuals face high levels of prejudice in interpersonal relationships. However, limited experimental research has examined the role of identity disclosure on anti-transgender prejudice.

**Methods:**

Drawing upon research on distrust and identity disclosure, two between-participants experiments (total n = 802) examined the role of intentional and unintentional identity disclosure on negative attitudes (Studies 1 & 2), perceived deception (Studies 1 & 2) and distrust (Study 2) toward two potentially concealable and historically distrusted identities (transgender and atheist). Specifically, the current studies examine the impact of a target’s stigmatized identity (transgender or atheist) and method of disclosure (intentional or unintentional) on perceptions of the target, perceived deceptiveness, and distrust toward the target.

**Results:**

Our findings demonstrated that compared to atheists, transgender targets elicited greater levels of prejudice and were viewed as more deceptive, and that this effect was amplified if the target did not intentionally reveal their identity. Study 2 demonstrated that perceived deception mediated the relationship between reveal type (i.e., intentional vs. unintentional) and prejudice toward participants who read about a transgender (but not atheist) target.

**Discussion:**

We discuss the implications of these findings for reducing prejudice toward binary transgender individuals, particularly those who do not voluntarily disclose their identity.

## Introduction

The number of people who identify as openly transgender (an overarching term used to describe individuals who do not identify with the gender they were assigned at birth) has increased rapidly over the last decade (Meerwijk and Sevelius, 2017). However, despite changes in visibility and frequency of open identification, public perceptions of transgender individuals remain negative (Doan et al., 2019). For example, in the United States, a record number of bills were proposed in 2021 that would limit access to healthcare, restrict transgender youth from participation in sports, or otherwise discriminate against transgender identities (PBS, 2021). This record was again exceeded in only the first 4 months of 2022, with the proposal of 238 anti-transgender bills across the US (Lavietes and Ramos, 2022). Moreover, although many hate crimes against transgender or gender non-conforming individuals go unreported, the Human Rights Campaign (HRC) labeled 2021 as the deadliest year on record for anti-transgender murders in the United States (Human Rights Campaign, 2021). Similarly, the United Kingdom has seen a surge in anti-transgender sentiment since 2017 (Vincent et al., 2020). A more thorough understanding of anti-transgender attitudes could aid in future methods of prejudice reduction toward this growing but stigmatized group.

Although there is a growing body of research examining anti-transgender attitudes, sexual minorities are most often used as a comparison group in this research (e.g., Nagoshi et al., 2008; Tebbe and Moradi, 2012; Norton and Herek, 2013; Prusaczyk and Hodson, 2019; Totton and Rios, 2021), a choice that may limit understanding of the various factors contributing to prejudice. While LGBT identities are often linked together in social discourse (e.g., the Queer community), it is empirically relevant to compare stigmatized groups, not only based on social groupings, but also based on the specific attitudes or emotions that drive prejudice toward them. For example, previous research has demonstrated that both transgender individuals (e.g., Totton and Rios, 2021) and atheists (Franks and Scherr, 2014) are perceived as untrustworthy, and elicit greater levels of distrust and prejudice, than gay men and lesbians. Despite eliciting similar emotions (distrust) and greater levels of prejudice than gay men and lesbians, atheists and transgender individuals have not previously been compared to one another in research.

Outside of being historically distrusted, both atheists and transgender identities are potentially concealable identities. While not all transgender individuals perceive themselves or are perceived by those around them as having a concealable identity, both binary (transgender individuals who identify as men or women) and nonbinary (transgender individuals who identify as neither a man nor woman, as both man and woman, or with another identity) identities are potentially concealable for portions of the transgender population. Although the choices and consequences of disclosure of stigmatized identities have been well studied from the perspective of the discloser (see Follmer et al., 2020 for review), research on disclosure expectations from the perspective of the recipient of a disclosure is far more limited. Previous research suggests that the choice to or not to disclose either a transgender (e.g., Law et al., 2011; Martinez et al., 2017; Fernandez and Birnholtz, 2019) or atheist (e.g., Abbott and Mollen, 2018; Abbott et al., 2020; Brewster et al., 2020; Abbott and Anaya, 2022) identity is often a difficult decision for the discloser, bearing many potential risks and benefits. Moreover, previous research suggests that both atheists and transgender individuals may face fears of stigma or negative responses when disclosures surround sensitive or high stakes interpersonal scenarios, such as romantic relationships (e.g., Fernandez and Birnholtz, 2019; Abbott et al., 2020; Abbott and Anaya, 2022). Given the dearth of literature on perceptions of disclosures, particularly toward transgender or atheist targets, the current study aims to explore perceptions of different types of disclosures of atheist and transgender identities in interpersonal dating scenarios.

To this end, the current research makes two key contributions to the existing literature. First, we explore attitudes toward two stigmatized and historically distrusted groups (transgender individuals and atheists) to further illuminate the emotions and attitudes that may drive negative attitudes toward members of these groups. Second, we expand on previous disclosure research to evaluate perceptions of disclosures of transgender or atheist identities under different circumstances. Specifically, we evaluate perceptions of intentional or unintentional disclosures of identity in the context of an interpersonal relationship. To our knowledge, the current studies are the first to explore the impact of unintentional (vs. intentional) disclosures of a stigmatized identity on perceptions of distrust and prejudice. We posit that both identity and disclosure work in tandem to predict negative attitudes and emotions toward the target. In the following sections, we review research on anti-atheist and anti-transgender attitudes, and on disclosure of stigmatized identities.

### Anti-transgender and anti-atheist attitudes

Previous research has examined individual differences predictive of anti-transgender attitudes, such as sexual orientation (Norton and Herek, 2013), gender (Nagoshi et al., 2008), traditional gender role attitudes, and need for closure (Tebbe and Moradi, 2012). When considering the psychological underpinnings of anti-transgender attitudes, some theoretical perspectives suggest that anti-transgender attitudes may stem from deviations to the gender binary and binary expectations surrounding gender presentation (Morgenroth and Ryan, 2021). Moreover, research has posited that while anti-transgender attitudes share many common factors with anti-lesbian, gay, or bisexual attitudes, anti-transgender attitudes are more negative (e.g., Norton and Herek, 2013) and have additional, distinct antecedents (Huffaker and Kwon, 2016). For instance, perceived deceptiveness is theorized to be unique to anti-transgender attitudes (Huffaker and Kwon, 2016). The notion that transgender individuals are viewed as being deceptive or “hiding” a portion of their identity has also been proposed in theoretical (e.g., Bettcher, 2007; Surinder, 2018; Billard, 2019), legal (e.g., Lee and Kwan, 2014; Sharpe, 2018) and qualitative (Wilchek-Aviad et al., 2020) domains. This argument has garnered some empirical support in recent research, which found that amongst cisgender heterosexual participants, distrust, and more specifically perceived deceptiveness, were important predictors of anti-transgender attitudes (Totton and Rios, 2021).

Given the role of distrust in anti-transgender attitudes, the current paper examines anti-transgender attitudes using a previously unused, but similarly distrusted comparison group, atheists. Despite holding distinctly different identities, atheists and transgender individuals are empirically interesting to compare for a variety of reasons. The percentages of people who openly identify as atheist and as transgender have more than doubled within the last decade, increasing awareness of both identities in mainstream society (Flores et al., 2016; Meerwijk and Sevelius, 2017; Pew Research, 2019). Despite increased identification, both groups continue to be perceived as highly untrustworthy (Gervais et al., 2011; Franks and Scherr, 2014; Huffaker and Kwon, 2016; Gervais et al., 2017; Grove et al., 2020) and face high levels of social stigma (Anderson, 2020; Rios et al., 2021). Moreover, both transgender and atheist identities are potentially concealable, yet they are deemed particularly important in dating or romantic contexts (e.g., Lipka and Martinez, 2014; Blair and Hoskin, 2019; Brown, 2022). Given the similarities in elicited emotions, the current study looks at perceptions of atheists and transgender individuals in highly sensitive interpersonal situations (i.e., dating) in which there may be a high expectation of disclosure of either identity.

We argue that this comparison is important, in part, because distinct outgroups may be distrusted for different reasons. For instance, biracial individuals who do not automatically disclose their identity are distrusted based on the perception that they are confused about their identity (Albuja et al., 2018), whereas bisexual individuals are distrusted based in part on a perception that they hold an “ambiguous” identity (Garelick et al., 2017). Although previous research has linked anti-transgender distrust to deceptiveness (Totton and Rios, 2021), and anti-atheist distrust to perceptions of being a “moral wildcard” (Gervais et al., 2011), it is possible that perceived deceptiveness is driven by the perception that an identity is being “hidden.” If this is the case, stigmatized identities that are historically distrusted may be similarly viewed as deceptive if they are perceived as being hidden, rather than intentionally revealed. As such, the current studies manipulate identity disclosure information to evaluate this possibility, thus further examining potential underpinnings of perceived deception and distrust. In the following sections, we highlight choices surrounding the disclosure of personal information as factors that may underlie perceived deceptiveness and feelings of distrust. Understanding the components and situational predictors of distrust sheds light on ways to increase trust of these groups in the future.

### Identity disclosure and deception

Research on disclosure of information surrounding stigmatized identities points to the important ramifications of disclosure both for individuals with a stigmatized identity and for their interaction partners. For stigmatized individuals, choices surrounding identity disclosure may have both psychological and social consequences (see Follmer et al., 2020, for a review). Indeed, within an organizational context, the decision to disclose personal information surrounding a stigmatized identity is considered to be one of the most difficult decisions faced by minorities in the workplace (Marrs and Staton, 2016). Recipients of disclosures similarly may view disclosures of identity as informative when considering their trust and connection with the discloser. Research on cross-race friendships found that self-disclosure surrounding identity was associated with greater trust and lower levels of prejudice toward outgroup members (Turner et al., 2007). However, to our knowledge, this research has not been expanded to potentially concealable stigmatized identities, such as transgender or atheist identities.

Perceptions of disclosure of potentially concealable identities are critical in understanding backlash against historically stigmatized groups who do not immediately disclose their identity. For example, in the United Kingdom, transgender individuals have been tried and convicted of “gender fraud,” a charge asserting that a person misrepresented or was intentionally deceptive about their gender identity, particularly in the context of romantic relationships (Sharpe, 2018). Similarly, the transgender panic defense, which hinges on the notion that a defendant lost control of their emotions upon learning the victim’s transgender identity, was still legal in 33 states as of 2021 (Lee and Kwan, 2014; The National LGBTQ+ Bar Association, 2021). These legal ramifications suggest that transgender individuals who do not instantly reveal their transgender identity are in some way culpable in the crimes committed against them (Trans* panic defense) or in the deception of another (gender fraud). As such, examining perceptions of disclosure toward this highly stigmatized community is an important step in understanding the undue onus placed on potentially concealable and distrusted communities to disclose their identity.

The current research evaluates perceptions of trust and deception when information about a stigmatized identity is unintentionally (i.e., accidentally) as opposed to intentionally revealed. We evaluate this in the context of transgender individuals and atheists, both of whom hold potentially concealable stigmatized identities, but may face different societal expectations surrounding the disclosure of their identity. Specifically, we evaluate perceptions of either transgender or atheist targets who personally (and intentionally) reveal their identity, or whose identity is “found out” through the discovery of personal information about the target.

### Overview of the current studies

Studies 1 and 2 used a first-date scenario to examine perceptions of transgender and atheist individuals’ deceptiveness and (in Study 2) general trustworthiness in the context of a potential close interpersonal relationship. We focused on the perceptions of cisgender, heterosexual participants who do not identify as atheists, to remove potential confounds in overlap of identity. We also examined perceptions of transgender or atheist individuals as deceptive about their identity when their identity is intentionally shared (by self-disclosure) as opposed to when their identity is accidentally revealed (by another person finding information that discloses their identity). If perceived deceptiveness of transgender individuals is related to perceptions that they have “withheld” critical personal information, transgender targets who do not self-disclose may be viewed as more deceptive than transgender targets who self-disclose. Similarly, atheists who withhold information may be viewed as more deceptive compared to targets (atheists or transgender) who openly reveal information. This distinction helps to elucidate the roles of target identity and information disclosure in perceived deceptiveness and prejudice.

Across two studies, we tested four hypotheses:

*Hypothesis 1*: There will be main effects of target group, such that transgender people will be viewed as more deceptive compared to atheists. Given that both groups are historically distrusted and have not been previously compared, we do not have an a priori hypothesis regarding trust.

*Hypothesis 2*: Given previous research demonstrating that both transgender individuals (e.g., Norton and Herek, 2013; Totton and Rios, 2021) as well as atheists (Franks and Scherr, 2014) face higher levels of prejudice than gay men and lesbians, we explore prejudice levels toward transgender individuals and atheists. Given rates of violence against transgender people (e.g., James et al., 2016), we anticipate that transgender targets will face higher levels of prejudice than atheist targets.

*Hypothesis 3*: Transgender and atheist individuals whose identities are accidentally revealed will be viewed as more deceptive (H3a) and more untrustworthy (H3b) than transgender and atheist targets who intentionally reveal their identity, as participants may feel as if the target’s identity has been “hidden” from them.

*Hypothesis 4*: There will be an interaction between target group (transgender vs. atheist) and disclosure condition (intentional or accidental reveal). Relative to all other conditions, the “transgender accidental reveal” condition will be subject to the greatest levels of deception due to both their identity (transgender) and their failure to disclose their identity (i.e., it was discovered by accident rather than intentionally revealed).

## Study 1

Study 1 used a 2 (participant gender) × 2 (atheist or transgender individual) × 2 (intentional or accidental identity reveal) design, leading to a total of 8 cells. Participant gender was included in the design because, in line with previous research, we anticipated that male participants would display greater levels of prejudice, distrust, and perceived deception than female participants. However, consistent with prior work on anti-transgender attitudes, we did not anticipate interactions between gender and experimental conditions (Totton and Rios, 2021).

### Methods

#### Participants

Four hundred and five Mechanical Turk Workers (216 women, 184 men, 5 transgender individuals) participated in exchange for compensation. Participants were randomly assigned to read a vignette about a transgender individual whose identity was accidentally revealed (*n* = 101), a transgender individual who intentionally revealed their identity (*n* = 100), an atheist individual whose identity was accidentally revealed (*n* = 103), or an atheist individual who intentionally revealed their identity (*n* = 101). When both atheist/agnostic individuals (*n* = 113) and non-heterosexual or non-cisgender individuals (*n* = 61) were removed from analysis, 252 participants remained (140 women, 112 men). A sensitivity analysis using G*Power 3.1 (Faul et al., 2009) was run for a 2 × 2 design, since we did not anticipate gender differences, to detect small-to-medium effects (*f* = 0.18) with 80% power and an alpha of.05.

#### Procedures and materials

After providing informed consent, participants responded to a series of demographic questions. Next, participants were randomly assigned to read one of four vignettes in which they went on a date with one of the following people: (1) a transgender person who intentionally revealed their transgender identity to the participant, (2) a transgender person whose identity was accidentally revealed to the participant, (3) an atheist person who intentionally revealed their atheist identity to the participant, or (4) an atheist person whose identity was accidentally revealed to the participant. The gender of the person participants read about was based on participants’ gender and self-reported romantic interest. However, since queer identified participants were excluded, the retained participants read about a participant who had a gender identity different from their own (i.e., male participants read about a trans or atheist female, female participants read about a trans or atheist male). This programming was intended to reduce the likelihood that participants’ interest or lack thereof might be based solely on the pronouns being used by the target, rather than the target’s identity or the interaction. Paired examples from the atheist unintentional reveal vignette and the transgender unintentional reveal vignette are provided below.

*Atheist Unintentional Reveal*

*You are at a bar with Taylor, whom you recently met on a dating site. You have been enjoying your conversation and have found that you have many similar interests and hobbies. Throughout the evening you discover that your movie and music preferences and even your favorite sports teams are extremely compatible. At one point in your conversation, Taylor mentions that she (he) has really enjoyed hanging out with you this evening and asks if you would be interested in meeting up again in the future.*

*You agree that you’ve had a great evening and would love to meet up again in the future. Your conversation continues, and you continue to have a great time full of engaging conversation with Taylor.*

*At the end of the night, Taylor heads to the bar to pay for her (his) drinks. As she (he) pulls out her (his) credit card another card falls to the floor. Taylor doesn’t seem to notice, but you reach down to pick it up. Picking up the card, you notice that it is a group membership card. The card states that it is a membership to “American Atheist”, a group dedicated strictly to Atheists. Scanning the card further, you notice that the card has Taylor’s name listed on it.*

*Transgender Unintentional Reveal*

*You are at a bar with Taylor, whom you recently met on a dating site. You have been enjoying your conversation and have found that you have many similar interests and hobbies. Throughout the evening you discover that your movie and music preferences and even your favorite sports teams are extremely compatible. At one point in your conversation, Taylor mentions that she (he) has really enjoyed hanging out with you this evening and asks if you would be interested in meeting up again in the future.*

*You agree that you’ve had a great evening and would love to meet up again in the future. Your conversation continues, and you continue to have a great time full of engaging conversation with Taylor.*

*At the end of the night, Taylor heads to the bar to pay for her (his) drinks. As she (he) pulls out her (his) credit card another card falls to the floor. Taylor doesn’t seem to notice, but you reach down to pick it up. Picking up the card, you notice that it is a driver’s license. The person in the photo is clearly Taylor, but the name on the identification is listed as Paul (Sarah). Scanning the card further, you notice that Paul (Sarah) is listed with all of the same physical characteristics of Taylor but is listed as a male (female) under sex.*

The intentional reveal vignettes read identically until the final paragraph, at which point Taylor shares their identity (atheist or transgender) with the participant. All vignettes can be found in the Appendix.

Participants then answered four questions about their attitudes toward the target they interacted with (e.g., “To what extent would you like to be friends with Taylor?”) on a 1 (*not at all*) to 7 (*very much)* Likert-type scale. These questions were averaged together to form a measure of target-specific prejudice (*M* = 5.34, SD = 3.11).

Next, participants were provided with definitions for either transgender or atheist (depending on their condition) as a reminder. A transgender individual was defined as “a person who identifies as a gender different from the one biologically assigned at birth,” and an atheist was defined as “a person who does not believe in the existence of a god or gods.” After reading those definitions, participants answered four questions about the extent to which they viewed the target as being confused (e.g., “Because Taylor is transgender [atheist] she [he] is still figuring out who he [she] is”; *M* = 3.68, SD = 1.64) as well as four questions about the extent to which they viewed the target as being deceptive (e.g., “It is dishonest for Taylor not to reveal his [her] atheist [transgender] identity to others”; *M* = 3.74, SD = 1.81), on a 1 *(not at all)* to 7 *(completely)* scale. Given that previous research has established that perceived deception, not perceived confusion, predicts distrust and prejudice toward transgender individuals, the confusion analyses are not a variable of focus and are therefore presented in the supplementary analyses.

Finally, all participants were debriefed and were provided information about the hypothesis of the study, as well as resources (e.g., websites) for learning more about LGBT identities. All materials can be viewed in the Appendix.

### Statistical analyses

Data from Study 1 were analyzed using SPSS to examine the impact of target person, disclosure condition, participant gender, and their interactions on perceptions of deception, distrust, and prejudice. Specifically, we conducted a series of 2 × 2 × 2 ANOVAs with post-hoc comparisons to examine main effects (i.e., of target person condition and disclosure condition) and interactions (i.e., between target person condition and disclosure condition). For each of these analyses, participant gender was included as a factor, as male participants tend to express greater levels of prejudice, distrust, and perceived deception toward transgender targets than do female participants (Totton and Rios, 2021; see also Tebbe and Moradi, 2012; Riggs and Sion, 2017). However, given that previous research only found main effects of participant gender (but not interactions) participant gender was not expected to interact with condition. Finally, since perceived confusion was included as a variable, we analyzed the results and included them in the Supplementary material.

#### Prejudice

##### Participant gender

There was not a significant effect of gender on attitudes toward the target (Male: *M* = 5.18, SE *=* 0.29, 95% CI [4.61, 5.74]; Female: *M* = 5.29, SE *=* 0.25, 95% CI [4.79, 5.79]), *F*(1, 244) = 0.09, *p* = 0.77, *η*^2^*_p_* < 0.001.

##### Atheist or transgender condition

Critically, the main effect of atheist or transgender condition was significant: Participants reported less positivity (more prejudice) toward transgender targets (*M* = 4.37, SE = 0.28, 95% CI [3.82, 4.92]) than toward atheist targets (*M* = 6.10, SE = 0.26, 95% CI = [5.58, 6.61]), *F*(1, 244) = 20.58, *p* < 0.001, *η*^2^_p_ = 0.078. These results support Hypothesis 2.

##### Intentional or accidental reveal conditions

Whether a transgender or atheist target’s identity was intentionally revealed (M = 5.49, SE = 0.27, 95% CI [4.95, 6.03]) or was accidentally revealed (*M* = 4.97, SE = 0.27, 95% CI [4.45, 5.50]*)* did not significantly impact prejudice toward the target, *F*(1, 244) = 1.83, *p* = 0.177, η^2^_p_ = 0.007.

##### Interactions

**Figure 1:**
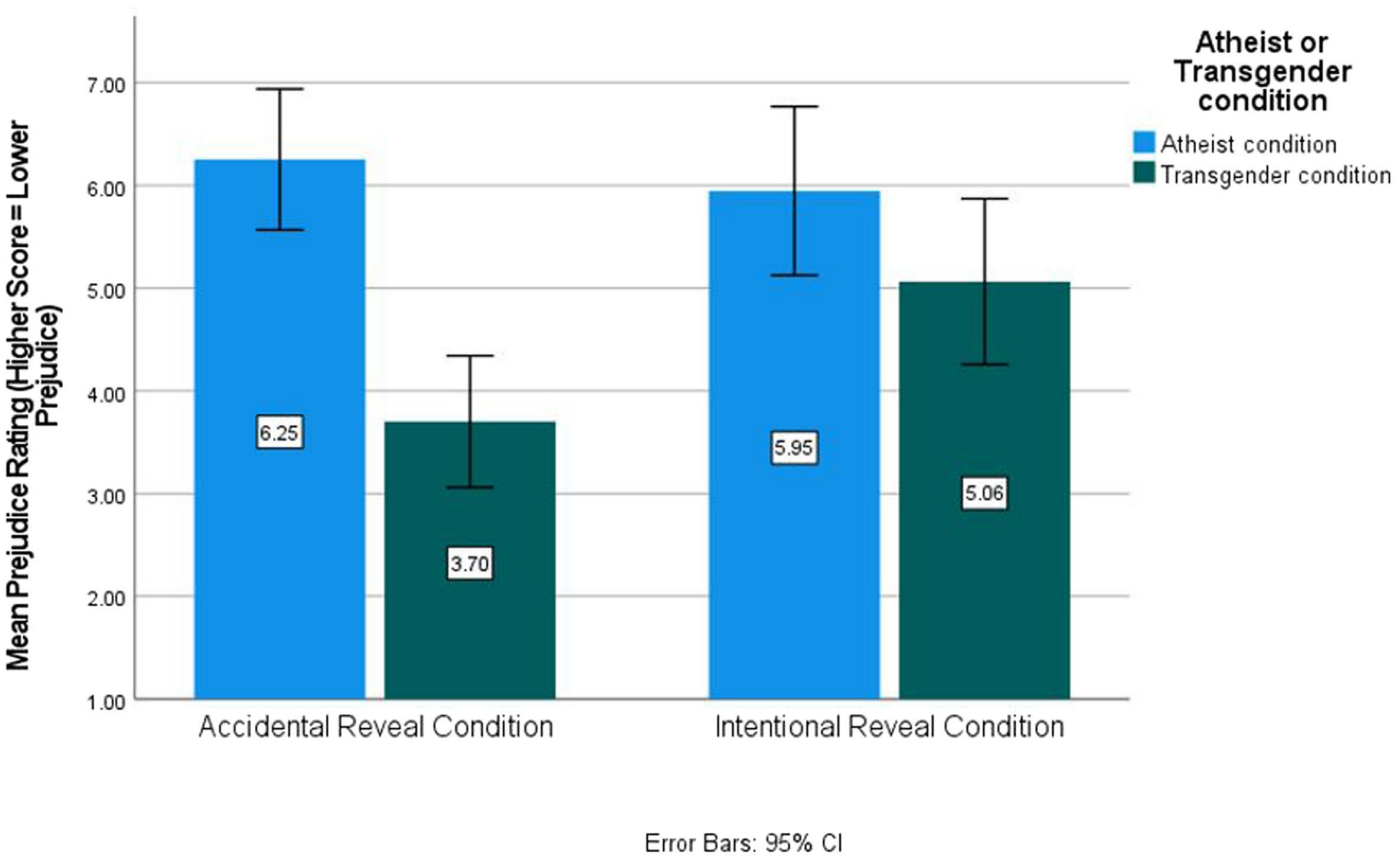
Target-specific attitudes by condition for Study 1 (lower score = higher prejudice).

The interaction between the atheist/transgender condition and the intentional or accidental reveal condition was significant, *F*(1, 244) = 5.38, *p* = 0.021, *η*^2^_p_ = 0.022. Post-hoc analyses indicated that transgender targets whose identity was accidentally revealed (*M* = 3.70, SE *= 0*.41, 95% CI [2.85, 4.48]) were not viewed significantly more negatively than transgender individuals who intentionally revealed their identity (*M* = 5.07, SE = 0.38, 95% CI [4.33, 5.81], *p =* 0.067) but were viewed more negatively than atheists whose identity was accidentally revealed (*M* = 6.25, SE = 0.34, 95% CI [5.61, 6.95], *p <* 0.001). Transgender targets who intentionally revealed their identity were not viewed significantly more negatively than atheists who intentionally revealed their identity (*M* = 5.95, SE = 0.40, 95% CI [5.13, 6.69], *p =* 0.122). This interaction is depicted in Figure 1.

The interaction between participant gender and the atheist/transgender condition was not significant, *F*(1, 244) = 3.27, *p* = 0.072, *η*^2^_p_ = 0.013.

#### Deception

##### Participant gender

There were no significant gender differences in perceptions of deception (male participants: *M* = 4.01, SE = 0.15, 95% CI = [3.72, 4.31], female participants: *M* = 3.63, SE = 0.13, 95% CI [3.37, 3.89]), *F*(1, 244) = 3.64, *p* = 0.058, *η*^2^_p_ = 0.015.

##### Atheist or transgender condition

Critically, the main effect of atheist or transgender condition was significant: Participants reported greater perceived deceptiveness toward transgender individuals (*M* = 4.68, SE = 0.15 CI [4.39, 4.97]) than toward atheist individuals (*M* = 2.96, SE = 0.14, 95% CI [2.69, 3.23]), *F*(1, 244) = 72.91, *p* < 0.001, *η*^2^_p_ = 0.230. This supports Hypothesis 1.

##### Intentional or accidental reveal condition

Whether a transgender or atheist target’s identity was intentionally revealed (*M* = 3.75, SE = 0.14, 95% CI [3.47, 4.03]) or was accidentally revealed (*M* = 3.90, SE = 0.14, 95% CI [3.62, 4.17]) did not significantly impact perceived deceptiveness, *F*(1, 244) = 0.52, *p* = 0.47, *η*^2^_p_ = 0.002, which did not support Hypothesis 3a.

##### Interactions

The interaction between atheist/transgender condition and intentional/accidental reveal condition was not significant, *F*(1,244) = 1.10, *p* = 0.296, *η*^2^_p_ = 0.004. The interaction between participant gender and the atheist/transgender condition was not significant, *F*(1, 244) = 3.27, *p* = 0.072, *η*^2^_p_ = 0.013.

### Study 1 discussion

Study 1 found that compared to atheists, transgender targets were viewed as more confused and more deceptive, and were rated more negatively (subject to greater prejudice). Whether targets accidentally or intentionally revealed their identity did not produce a significant main effect in Study 1. Of interest, though, an interaction effect between atheist/transgender condition and reveal condition showed that transgender individuals whose identity was intentionally revealed were viewed less negatively than those whose identity was accidentally revealed. These results suggest that transgender individuals may be held to a higher expectation to disclose personal information, and that failure to do so may elicit greater levels of prejudice. However, we did not find an interaction between atheist/transgender condition and reveal condition on perceived deceptiveness. We suspected that the wording of many of our questions (e.g., “Because Taylor is transgender [atheist]…) may have impacted participants’ interpretation of our questions. As such, we reworded these questions in Study 2 to decrease ambiguity.

## Study 2

Study 2 expanded upon the results of Study 1 in several ways. Unlike in Study 1 where the demographic option of atheist/agnostic was combined, participants in Study 2 were asked specifically whether they identified as atheist or agnostic, and only atheists were excluded from data analysis. Moreover, the perceived deception questions were slightly altered to address the potential that the wording of the questions (e.g., “Because Taylor is transgender…) may have impacted participants’ responses. In Study 2, we instead worded the questions to focus on the knowledge gained in the vignette (e.g., “Learning that Taylor is transgender…”). The questions used in both Study 1 and Study 2 can be found in the Appendix. Finally, to further evaluate perceived trust (Hypotheses 1c and 2b), participants were asked about the extent to which they trusted the target (in addition to questions about perceived deception).

### Methods

#### Participants

Three hundred and ninety-seven Prolific Workers (175 women, 221 men, 1 gender neutral individual) participated in exchange for compensation. Participants were randomly assigned to read a vignette about a transgender individual whose identity was accidentally revealed (*n* = 99), a transgender individual who intentionally revealed their identity (*n* = 97), an atheist individual whose identity was accidentally revealed (*n* = 100), or an atheist individual who intentionally revealed their identity (*n* = 101). When atheist (*n* = 23), non-heterosexual (*n* = 7), and non-cisgender participants (*n* = 1) were removed from analysis, 366 participants remained (161 women, 205 men). A sensitivity analysis using G*Power 3.1 (Faul et al., 2009) revealed that a sample of 386 in a 2 × 2 × 2 design was necessary to detect a medium effect (*f* = 0.25) with 80% power and an alpha of.05. Although we collected more participants than this threshold, the study was slightly underpowered after excluding non-cisgender or atheist participants.

#### Procedures and materials

All procedures and materials were identical to Study 1 with three exceptions. First, whereas participants in Study 1 responded to four questions about their desire to interact with or befriend the target, participants in Study 2 answered three semantic differential questions about their general attitudes toward the target (e.g., “On the whole, I think Taylor is”: 1 (*bad*) to 9 (*good*)). Second, the deception questions were slightly reworded to remove potential ambiguity and to emphasize the role of the dating disclosure rather than exclusively the target’s identity (e.g., “*If I knew Taylor in my childhood and she (he) told me she (he) was a transgender woman (man, atheist), I would feel like I did not know her (him) at all now.”* was changed to “*Learning that Taylor is transgender made me question everything (s) he told me about herself (himself) on our date”*). Finally, to rate participants’ perceptions of overall trust rather than just perceived deceptiveness, participants were asked to rate their perception of the target’s traits (*“On the whole I think Taylor is…”)*. Of interest were three traits related to trust: *trustworthy* (reverse scored), *dishonest*, and *a liar.* Similar to Study 1, analyses for the confusion variable as well as all materials are presented in the Appendix.

### Statistical analyses

All analyses were identical to study 1.

#### Prejudice

##### Participant gender

There was not a significant effect of gender on target-specific attitudes (male: *M* = 6.49, SE = 0.12, 95% CI [6.26, 6.72]; female: *M* = 6.43, SE = 0.13, 95% CI [6.12, 6.69]), *F*(1, 358) = 0.11, *p* = 0.74, *η*^2^*_p_* = 0.009.

##### Atheist or transgender condition

Critically, the main effect of atheist or transgender condition was significant: Participants reported less positivity (more prejudice) toward transgender targets (*M* = 5.89, SE = 0.13, 95% CI [5.65, 6.14]) than toward atheist targets (*M* = 7.02, SE = 0.12, 95% CI [6.79, 7.27]), *F*(1, 258) = 42.54, *p* < 0.001, *η*^2^_p_ = 0.105. This finding is in line with Hypothesis 2.

##### Intentional or accidental reveal conditions

Unlike in Study 1, participants in the intentional reveal conditions (*M* = 6.23, SE = 0.12, 95% CI [5.99, 6.48]) had more negative attitudes toward the target than participants in the accidental reveal conditions (*M* = 6.69, SE = 0.12, 95% CI [6.45, 6.93]), *F*(1, 358) = 6.83, *p* = 0.009, *η*^2^_p_ = 0.019.

##### Interactions

Unlike in Study 1, there was not a significant interaction between the atheist/trans condition and the intentional or accidental reveal condition, *F*(1, 358) = 0.53, *p* = 0.46, *η*^2^*_p_* = 0.002. There was a significant interaction between participant gender and the transgender/atheist condition, *F*(1,358) = 17.82, *p* < 0.001, *η*^2^_p_ = 0.047. Simple effects tests indicated that men in the transgender condition (*M* = 5.56, SE = 0.17, 95% CI [5.23, 5.89]) expressed higher rates of prejudice than men in the atheist condition (*M* = 7.42, SE = 0.16, 95% CI [7.11, 7.74], *p* < 0.001) or women in the transgender condition (*M* = 6.24, SE = 0.19, 95% CI [5.87, 6.60], *p* = 0.007).

#### Distrust

##### Participant gender

There was no significant difference in distrust between male (*M* = 3.02, SE = 0.09, 95% CI [2.85, 3.19]) and female (*M* = 2.92, SE = 0.96, 95% CI [2.74, 3.11]) participants, *F*(1, 358) = 0.53, *p* = 0.47, *η*^2^_p_ = 0.001.

##### Atheist or transgender condition

The main effect of atheist or transgender condition was significant, such that participants in the transgender condition (*M* = 3.61, SE = 0.09, 95% CI [3.42, 3.79]) reported higher levels of distrust than participants in the atheist condition (*M* = 2.32, SE = 0.09, 95% CI [2.15, 2.51]), *F*(1, 358) = 14.98, *p* < 0.001, *η*^2^_p_ = 0.219.

##### Intentional or accidental reveal conditions

Participants in the accidental reveal condition (*M* = 3.35, SE = 0.09, 95% CI [3.17, 3.52]) expressed significantly higher rates of distrust than participants in the intentional reveal condition (*M* = 2.60, SE = 0.09, 95% CI [2.42, 2.77]), *F*(1,358) = 32.25, *p* < 0.001, *η*^2^_p_ = 0.087, supportive of Hypothesis 3a.

##### Interactions

**Figure 2:**
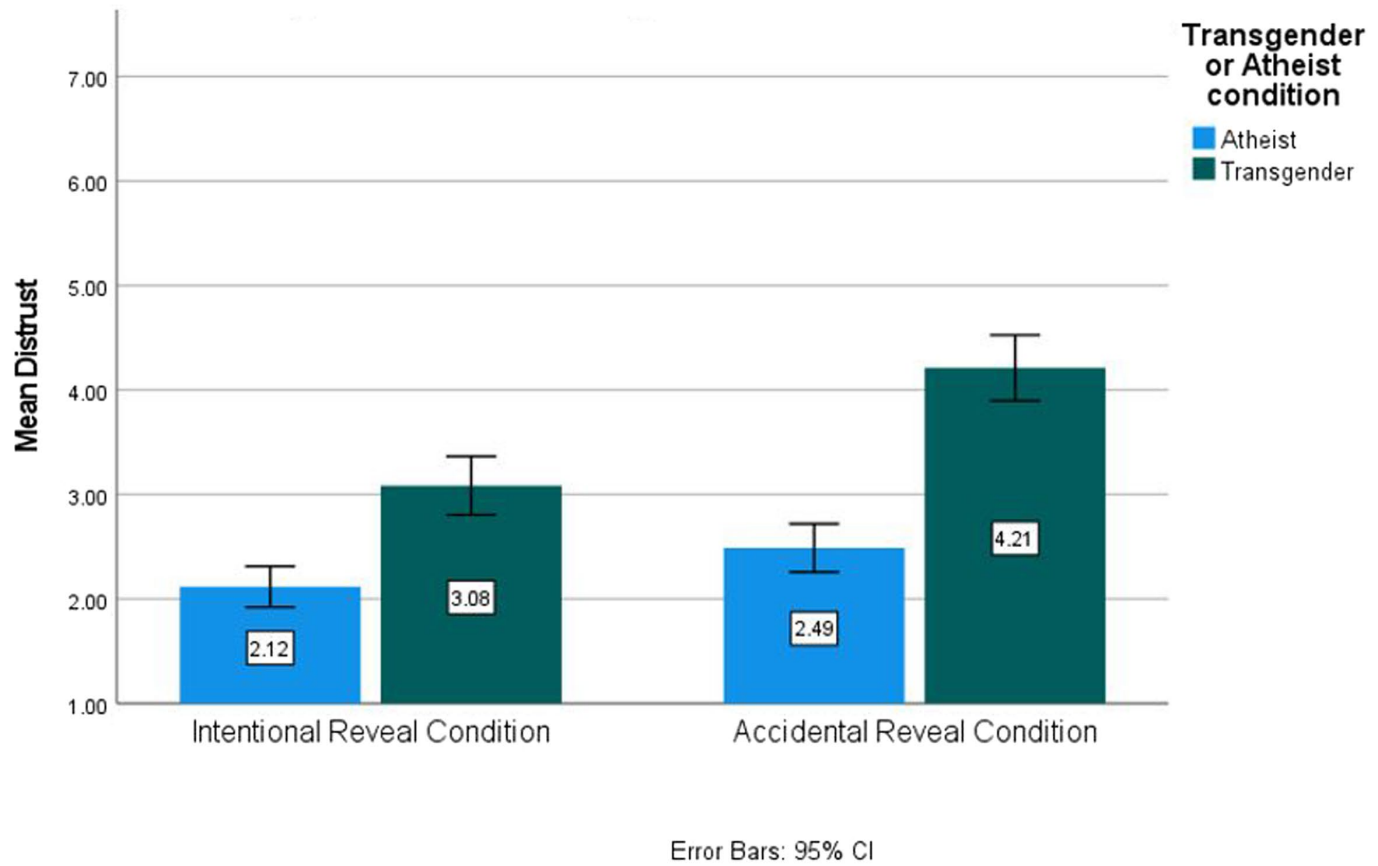
Distrust by condition for Study 2.

Importantly, there was a significant interaction between the transgender/atheist condition and the reveal condition, *F*(1,358) = 9.44, *p* = 0.002, *η*^2^_p_ = 0.026. Participants in the transgender accidental reveal condition (*M* = 4.12, SE = 0.13, 95% CI [3.93, 4.44]) expressed significantly higher rates of distrust than in the transgender intentional reveal condition (*M* = 3.04, SE = 0.13, 95% CI [2.78, 3.30], *p* < 0.001) or the atheist accidental reveal condition (*M* = 2.51, SE = 0.13, 95% CI [2.26, 2.76], *p* < 0.001). This supports Hypothesis 4 and is depicted in Figure 2.

There was also a significant interaction between participant gender and the trans/atheist conditions, *F*(1, 358) = 16.21, *p* < 0.001, *η*^2^_p_ = 0.043. Simple effects tests indicated that men in the trans condition (*M* = 3.92, SE = 0.12, 95% CI [3.68, 4.16]) expressed significantly higher rates of general distrust than men in the atheist condition (*M* = 2.12, SE *= 0*.12, 95% CI [1.89, 2.35], *p* < 0.001) or women in the trans condition (*M* = 3.31, SE = 0.14, 95% CI [3.04, 3.58], *p* < 0.001).

#### Deception

##### Participant gender

There were no significant gender differences in ratings of deception (male participants: *M* = 3.39, SE = 0.10, 95% CI [3.19, 3.59], female participants: *M* = 3.18, SE = 0.12, 95% CI [2.93, 3.38]), *F*(1, 358) = 2.22, *p* = 0.137, *η*^2^_p_ = 0.006.

##### Atheist or transgender condition

Supporting Hypothesis 1, the main effect of atheist or transgender condition was significant: Participants reported greater perceived deceptiveness toward transgender individuals (*M* = 4.28, SE = 0.11, 95% CI [4.06, 4.50]) than toward atheist individuals (*M* = 2.26, SE = 0.11, 95% CI [2.05, 2.50]), *F*(1, 358) = 171.10, *p* < 0.001, *η*^2^_p_ = 0.323.

##### Intentional or accidental reveal conditions

Supporting Hypothesis 3a, the main effect of the reveal condition was significant: Targets whose identity was accidentally revealed (*M* = 3.48, SE = 0.11, 95% CI [3.26, 3.69]) were viewed as more deceptive than targets whose identity was intentionally revealed (*M* = 3.10, SE = 0.11, 95% CI [2.85, 3.28]), *F*(1, 358) = 7.10, *p* = 0.008, *η*^2^_p_ = 0.019.

##### Interactions

**Figure 3:**
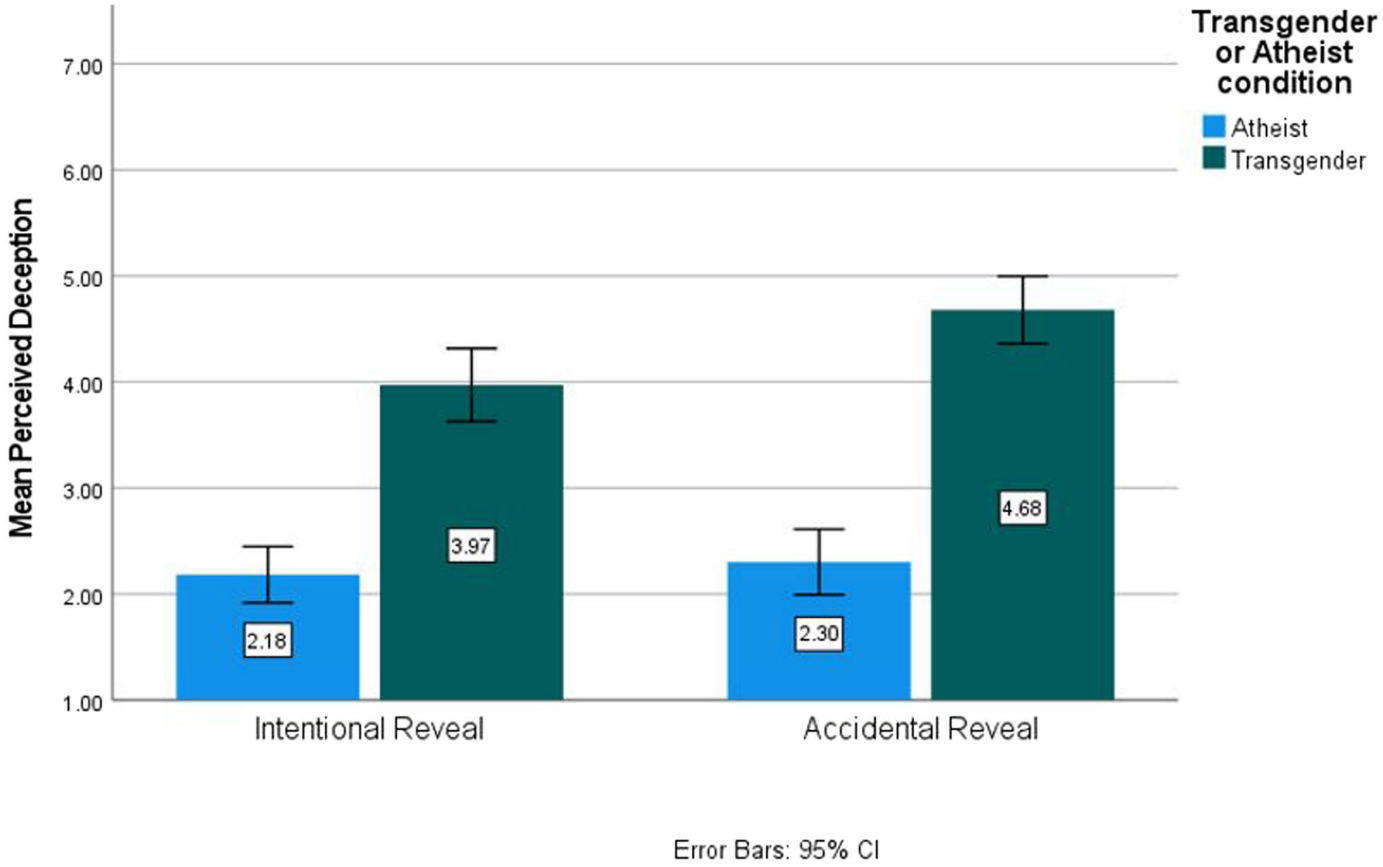
Desception by condition for Study 2.

There was a significant interaction between transgender/atheist condition and reveal condition, *F*(1,358) = 4.00, *p* = 0.046, *η*^2^_p_ = 0.011. Simple effects tests indicated that participants in the transgender accidental reveal condition (*M* = 4.64, SE = 0.16, 95% CI [4.33, 4.94]) expressed significantly higher perceptions of deception than participants in the transgender intentional reveal condition (*M* = 3.92, SE = 0.16, 95% CI [3.61, 4.29], *p* < 0.001) or participants in the atheist accidental reveal condition (*M* = 3.32, SE = 0.15, 95% CI [2.01, 2.62], *p* < 0.001). This interaction supports Hypothesis 4 and is depicted in Figure 3.

Moreover, there was a significant interaction between participant gender and the transgender/atheist condition, *F*(1, 358) = 13.87, *p* < 0.001, *η*^2^_p_ = 0.037. Simple effects tests indicated that men in the transgender condition (*M* = 4.68, SE = 0.15, 95% CI [4.39, 4.97]) expressed significantly higher perceptions of deceptiveness than men in the atheist condition (*M* = 2.09, SE = 0.14, 95% CI [2.12, 2.75], *p* < 0.001) and women in the transgender condition (*M* = 3.88, SE = 0.17, 95% CI [3.55, 4.20], *p* < 0.001).

### Mediated moderation analysis

**Figure 4:**
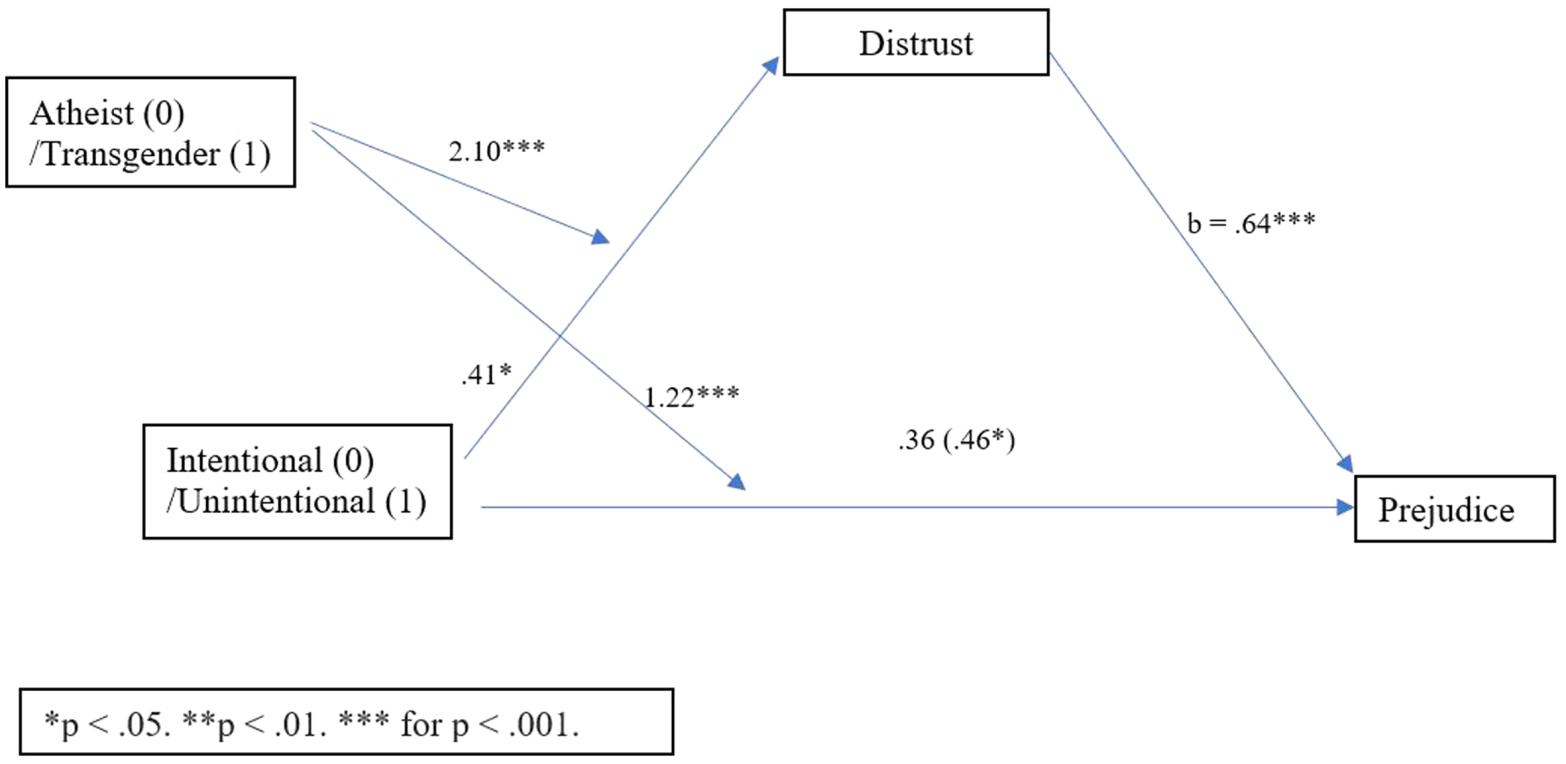
Process Model 8 moderated mediation with distrust Study 2.

Next, we evaluated the possibility that transgender individuals may be held to a higher expectation of disclosure than atheists, using a mediated moderation analysis. To test whether distrust mediated the relationship between reveal condition (predictor) and prejudice (outcome) for participants based on reading about a transgender or atheist identity (moderator), a mediated moderation analysis was conducted using PROCESS model 8 with 5,000 bootstrapping estimates (Hayes, 2013). This analysis revealed an overall indirect effect (*b* = −0.67, SE = 0.23, 95% CI [−1.13, −0.22]), which was significant among participants in the atheist condition (*b* = −0.86, SE = 0.16, 95% CI [−1.19, −0.55]), but also significant and stronger among participants in the transgender condition (*b* = −1.53, SE = 0.21, 95% CI [−1.94, −1.13]). This mediated moderation is depicted in Figure 4.

**Figure 5:**
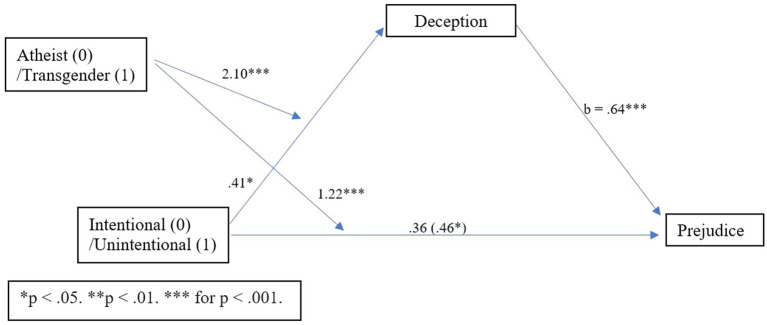
Process Model 8 moderated mediation with deception Study 2.

Next, to test whether deception mediated the relationship between reveal condition (predictor) and prejudice (outcome) based on reading about a transgender or atheist identity (moderator), a mediated moderation analysis was conducted using PROCESS model 8 with 5,000 bootstrapping estimates (Hayes, 2013). This analysis revealed an overall indirect effect (*b* = −0.36, SE = 0.19, 95% CI [−0.75, −0.02]), which was significant among participants in the transgender condition (*b* = −0.44, SE = 0.15, 95% CI [−0.74, −0.16]), but not among participants in the atheist condition (*b* = −0.07, SE = 0.13, 95% CI [−0.32, 0.18]). This mediated moderation is depicted in Figure 5.

### Study 2 discussion

The results of Study 2 replicated those of Study 1 in demonstrating that transgender targets are perceived as more deceptive and elicit greater levels of prejudice than atheist targets. Building upon prior studies, Study 2 also demonstrated that perceived distrust and deceptiveness may be amplified for transgender (but not atheist) individuals who do not disclose their identity. However, the interaction observed for prejudice in Study 1 was not observed in Study 2. Although more research is needed, one possible explanation involves the different prejudice measures used in Studies 1 and 2. Specifically, the prejudice measures used in Study 2 (semantic differential scales) may have been less effective at identifying prejudice toward individual targets than the target-specific questions used in Study 1. Additionally, Study 2 found interactions between gender and condition that were not observed in Study 1. Although these interactions were not anticipated, they suggest that effects may in part be due to negativity toward transgender women, particularly among cisgender heterosexual men. However, given that female participants read about a transgender or atheist man, and male participants read about a transgender or atheist woman, more research is needed to clearly understand the role of participant gender in Study 2. Finally, Study 2 demonstrated that both deception and distrust separately mediated the relationship between the reveal condition and prejudice when the transgender or atheist condition was included as a moderator. This suggests that both perceived deceptiveness in particular and feelings of distrust in general play important roles in anti-transgender prejudice.

## General discussion

The current research examined prejudice, deception, and distrust (Study 2) toward atheist or transgender targets whose identity was intentionally or unintentionally revealed. Across both studies, transgender individuals were viewed as more deceptive and elicited greater prejudice compared to atheists. Importantly, Study 1 found that transgender, but not atheist, targets whose identity was unintentionally revealed elicited greater prejudice. Although this effect was not found for prejudice in Study 2, a similar interaction was observed for both distrust and deception, each of which had indirect effects on prejudice. It is possible that changes in the wording of the prejudice measures between Study 1 and 2 can account for differences in the interaction effects between Studies 1 and 2. Critically, however, a mediated moderation model conducted in Study 2 found that both distrust and deception separately mediated the relationship between reveal condition and prejudice for participants in the transgender condition. Taken together, the current studies provide evidence that transgender individuals may be held to particularly high expectations of disclosure, and that perceived failures to disclose may be associated with greater prejudice (Study 1) or greater distrust and perceived deceptiveness (Study 2).

Understanding expectations of disclosure for transgender individuals, who may be perceived by others as holding a concealable identity, is particularly important given that as of 2021, the “transgender panic defense” (the legal argument that a defendant became emotionally overwhelmed at learning a transgender person’s identity and acted uncontrollably, typically with violence as a result) was only outlawed in only 16 states and the District of Columbia (The National LGBTQ+ Bar Association, 2021). Similarly, in the United Kingdom, “gender fraud” charges have been used to prosecute transgender individuals, particularly transgender youth, for perceived failure to disclose their transgender identity (Sharpe, 2018). The existence of panic defenses or gender fraud charges subtly places the onus onto transgender people to constantly and immediately disclose their identity to potential dating partners, a task that may be unrealistic as well as mentally and emotionally taxing (e.g., Brumbaugh-Johnson and Hull, 2019). Importantly, however, while failure to disclose a transgender identity may elicit greater levels of prejudice, distrust, and perceived deceptiveness, there was a main effect (not only interaction effects) of the transgender condition, suggesting that in the context of dating relationships, even open disclosure of a transgender identity may not be enough to fully offset perceptions of distrust, deceptiveness, or prejudicial attitudes. This suggests that placing the onus for identity disclosure on transgender individuals is unlikely to be an effective means of decreasing anti-transgender prejudice in the context of cisgender heterosexual interpersonal relationships. Moving forward, this research points to the need to deemphasize expectations of disclosure for transgender individuals, as constant and continuous identity disclosure is not only unrealistic, but also fails to resolve misconceptions that transgender individuals are being deceptive about their identity. Rather, prejudice reduction efforts may be more effective by reducing perceptions of deceptiveness directly.

Study 2 revealed unanticipated interaction effects between participant gender and the atheist/transgender condition. Although previous research has suggested that males respond more negatively on attitudes and prejudice measures more generally (e.g., Talley and Bettencourt, 2008; Tebbe and Moradi, 2012; Riggs and Sion, 2017; Totton and Rios, 2021) we did not anticipate that there would be interaction effects with the based on the reveal condition. However, our results demonstrated that in Study 2, cisgender heterosexual men expressed the greatest level of negativity toward transgender women. These results are potentially important in considering that violence is most frequently enacted upon transgender women, and more specifically, Black transgender women (James et al., 2016). The differences found in interactions may be due to sample size differences or differences in wording of the measures. It is possible that changes in wording of the prejudice or deception measures were more explicit and led to differences of reaction in cisgender heterosexual male participants. However, the results of Study 2 highlight the need to further evaluate these interaction effects, as the responses of cisgender heterosexual males may be critical to focus on from a prejudice reduction perspective. Indeed, previous work has demonstrated that perceived threats to masculinity are related to greater levels of sexual and gender prejudice amongst male participants (e.g., Talley and Bettencourt, 2008; Konopka et al., 2021). Transgender women may pose a greater threat to masculinity than atheists, particularly in the context of a dating vignette, and as such, this may have driven the gender-based differences observed in Study 2. Future research examining the role of participant gender should explicitly examine threats to masculinity to illuminate this potentially important relationship.

This research also helps clarify previous work on the emotions and perceptions that drive anti-transgender prejudice. By manipulating both identity (transgender or atheist) and the disclosure of identity (intentional or unintentional reveal), the current research provides additional insight into the role of deception and general distrust in anti-transgender prejudice. Specifically, although previous work demonstrated a link between anti-transgender attitudes and perceived deception and distrust (Totton and Rios, 2021), the context of identity and identity disclosure had yet to be explored. The current research points to the effect not only of identity on perceived deception and distrust, but also of the means through which an identity is learned. This adds to an understanding of the factors that impact feelings of distrust or deception. Moreover, whereas previous research had only examined perceptions of distrust or deception from a theoretical lens (e.g., Huffaker and Kwon, 2016) or using identity-based labels such as “transgender person” (e.g., Totton and Rios, 2021), the current research takes the important step of using the more realistic paradigm of vignettes to understand attitudes based on both behavior and identity. However, while vignettes may provide more external validity than labels alone, they may not be representative of participants’ real behaviors upon having an undisclosed stigmatized identity disclosed in their day to day lives (e.g., Bauman et al., 2014; Eifler and Petzold, 2019). Indeed, it is possible that the disclosure of the transgender condition, in particular, may have elicited a stronger emotional response from participants given the use of the reveal/disclosure of transgender characters in media as a “shock” factor (Bettcher, 2007; Serano, 2016; Thach, 2021). If this is the case, the results may speak to the harmful nature of such “shock” revelations in media. Future research should seek to better understand participants’ behaviors and attitudes surrounding identity disclosure in more realistic settings and under scenarios that disclose the target’s identity in more subtle or nuanced means.

In the context of the vignettes used, it is possible that disclosures surrounding transgender identities are viewed differently from disclosures of other identities. While atheists are also a historically distrusted group, it is possible that failure to disclose an atheist identity is viewed as less egregious than failure to disclose information surrounding one’s gender or sexuality. Although the dating scenario was selected based on research that both atheist and transgender identities are viewed as highly important in dating contexts (e.g., Lipka and Martinez, 2014; Blair and Hoskin, 2019; Brown, 2022), it is possible that transgender identity is viewed as a more critical piece of information in early dating scenarios. Follow-up research should evaluate explicit expectations for disclosing various stigmatized identities (e.g., sexual orientation), as well as non-stigmatized identities to create a more nuanced understanding of the level of stigma faced by both transgender individuals and by atheists. Additionally, further research should further explore expectations of disclosure for transgender targets more specifically in contexts outside of dating relationships, as it is possible that expectations surrounding disclosure differ based on the context of the relationship.

While the current study evaluated perceptions of a transgender or atheist target more specifically, future research should expand this construct to examine whether negativity toward a singular transgender or atheist target extends to the broader group. The finding from the current studies speak to the potential of negative attitudes toward singular transgender individuals, and particularly those who do not disclose their identity in dating contexts. However, the potential that this expands to perceptions of the community at large (e.g., transgender or atheist individuals as a collective) is important to evaluate in the future as support for this would indicate a homogeneity in stereotypes and associated prejudices toward these stigmatized communities.

It is also critical to note that the current research only encompasses binary transgender identities that may be perceived as “passing” (i.e., likely to be perceived by others as the gender they identify with). As such, it does not consider perceptions of people with more “visible” transgender identities, who may still face difficult decisions surrounding explicit and intentional disclosure. Indeed, the act of passing alone may impact perceptions of deceptiveness of transgender individuals (e.g., Billard, 2019), and it is thus possible that transgender individuals perceived as holding a more visible identity may face different or distinct forms of societal pushback or stigma. Although many factors predict transgender individuals’ choices surrounding visibility and passing intentions (Anderson et al., 2020), further research comparing disclosure expectations based on visibility would be informative.

Moreover, the current research excludes nonbinary transgender individuals. Indeed, some research suggests that while prejudice toward both binary and nonbinary individuals may be driven by factors such as RWA, traditional masculinity, and anti-egalitarianism, traditional femininity is associated with more positive attitudes toward nonbinary individuals, but not toward binary transgender individuals (Perez-Arche and Miller, 2021). In conjunction, these results suggest that while binary and nonbinary transgender prejudice may share many commonalities, they are not synonymous, and the evaluation of nonbinary identities would contribute to a more comprehensive understanding of anti-transgender prejudice.

Although the current research is informative of perceptions toward transgender and atheist targets in the context of a potential dating relationship, this research did not include perceptions of the target as being immoral or morally unpredictable. While this is a domain that has largely already been applied to anti-atheist attitudes, it is possible that perceived moral violations also predict anti-transgender attitudes and therefore should be explored in future research. Moreover, atheists may be more distrusted or viewed as more deceptive under contexts other than dating. For example, distrust toward atheists may be more prominent in circumstances that elicit concerns of the morality or moral predictability of the target (e.g., trust in a target tasked with making a moral decision). While value is placed behind the morality of relational partners in long-term dating relationships, participants may place less value behind morality in short-term relationships (such as the vignette provided to participants) decreasing the impact of perceived immorality of atheist targets (Brown, 2022). Follow up research explicitly evaluating perceptions of atheist or transgender targets tasked with moral decisions would not only provide further clarity on distrust toward transgender and atheist individuals, but would allow for an exploration of anti-atheist attitudes in political domains in which atheist candidates would be entrusted with critical moral decisions.

The current research adds to previous research on the impacts of distrust and identity on prejudice by highlighting the role of both identity (e.g., transgender or atheist), as well as situations surrounding disclosure (e.g., intentionality) in predicting prejudice, even among historically distrusted groups. Indeed, perceptions of deception play a critical role in anti-transgender prejudice, and the current research highlights the compounding effects of disclosure (or lack thereof) on perceived deceptiveness. Importantly, the current research highlights that identity disclosure alone was not a sufficient method of decreasing perceptions of deceptiveness of transgender individuals. Given the continued acceptance of the transgender panic defense and numbers of anti-transgender legislation that have recently been proposed, the current research further points to the urgency of identifying effective methods of prejudice reduction. More specifically, this research highlights that effective prejudice reduction interventions may need to directly or indirectly focus on increasing perceived trust and believability of transgender identities.

## Data availability statement

The raw data supporting the conclusions of this article will be made available by the authors, without undue reservation.

## Ethics statement

The studies involving human participants were reviewed and approved by Amherst College Ohio University. The patients/participants provided their written informed consent to participate in this study.

## Author contributions

RT contributed to conceptualizing all studies, organized the data, performed the statistical analysis, and wrote and prepared the first draft of the manuscript. KR contributed to conceptualizing all studies, to data interpretation and revisions of the manuscript. NS contributed to the data organization for Study 2. All authors contributed to the article and approved the submitted version.

## Funding

This research was supported by RT’s institution through internal research funding.

## Conflict of interest

The authors declare that the research was conducted in the absence of any commercial or financial relationships that could be construed as a potential conflict of interest.

## Publisher’s note

All claims expressed in this article are solely those of the authors and do not necessarily represent those of their affiliated organizations, or those of the publisher, the editors and the reviewers. Any product that may be evaluated in this article, or claim that may be made by its manufacturer, is not guaranteed or endorsed by the publisher.
