# Supplementary material for: Distrusted disclosures: Deception drives anti-transgender but not anti-atheist prejudice
Source: Front Psychol. 2023 Jan 26;13:1006107. doi: 10.3389/fpsyg.2022.1006107 (PMC9908580; doi:10.3389/fpsyg.2022.1006107)
Supplement: Supplementary file 1 [file Data_Sheet_1.pdf]

Appendix

Vignettes for both Study 1 & 2

Instructions: *Please imagine the following situation specifically and as if it actually occurred. We will ask you to reflect on the details of the situation as well as the details of the story, so please pay close attention and fully consider the story as if it were truly happening to you.*

You are at a bar with Taylor, whom you recently met on a dating site. You have been enjoying your conversation and have found that you have many similar interests and hobbies. Throughout the evening you discover that your movie and music preferences and even your favorite sports teams are extremely compatible. At one point in your conversation, Taylor mentions that she (he) has really enjoyed hanging out with you this evening and asks if you would be interested in meeting up again in the future.

You agree that you've had a great evening and would love to meet up again in the future. Your conversation continues, and you continue to have a great time full of engaging conversation with Taylor.

From here the stories diverge based on condition and participant gender:

- Identity-revealed transgender condition
  - At the end of the night, Taylor heads to the bar to pay for her (his) drinks. As she (he) pulls out her (his) credit card another card falls to the floor. Taylor doesn't seem to notice, but you reach down to pick it up. Picking up the card, you notice that it is a driver's license. The person in the photo is clearly Taylor, but the name on the identification is listed as Paul (Sarah). Scanning the card further, you notice that Paul (Sarah) is listed with all of the same physical characteristics of Taylor but is listed as a male (female) under sex.
- Identity-revealed atheist condition
  - At the end of the night, Taylor heads to the bar to pay for her (his) drinks. As she (he) pulls out her (his) credit card another card falls to the floor. Taylor doesn't seem to notice, but you reach down to pick it up. Picking up the card, you notice that it is a group membership card. The card states that it is a membership to "American Atheist", a group dedicated strictly to Atheist. Scanning the card further, you notice that the card has Taylor's name listed on it.
- Self-disclosure transgender condition
  - Taylor says she (he) has something important to share with you before your evening is over. At this point, Taylor shares with you that she (he) is a transgender woman (man). Taylor explains that even though she (he) was raised as a male (female), she (he) is a woman (man).
- Self-disclosure atheist condition
  - Taylor says she (he) has something important to share with you before your evening is over. At this point, Taylor shares with you that she (he) is an atheist. Taylor explains that even though she (he) was raised religious, she (he) no longer has any belief in God or a higher power.

Instructions: *Please imagine how you would feel if this situation actually occurred. We ask that you answer the following questions as honestly and accurately as you are able to. Your responses will not be connected to you.*

Demographics for all studies

1. How do you define your gender? *(male, female, transgender male, transgender female, gender neutral/gender ambiguous, other)*
2. How do you define your sexual orientation? *(straight, gay, lesbian, bisexual, pansexual, asexual, other)*
3. What is your religious affiliation (Study 1: *Christian (Protestant), Christian (Catholic), Christian (other), Muslim, Jewish, Hindu, Buddhist, Agnostic/Atheist, another option not listed here*; Study 2: *Christian (Protestant), Christian (Catholic), Christian (other), Muslim, Jewish, Hindu, Buddhist, Agnostic, Atheist, another option not listed here*)
4. What is your political orientation? *(1 = extremely liberal, 7 = extremely conservative)*
5. What is your age?

Study 1 Questions

Prejudice.

Instructions: Please respond to the questions with the situation you just read in mind from 1 (*not at all*) to 7 (*very much*). Please imagine how you would feel and how you would respond. Please respond as honestly and accurately as you are able to. Your responses will not be connected to you.

1. To what extent would you like to go out on another date with Taylor?
2. To what extent would you like to be friends with Taylor?
3. To what extent do you like Taylor?
4. To what extent do you feel warmth toward Taylor?

Confusion and Deception

Instructions: Please indicate the extent to which you agree with each of the following statements from 1 (not at all) to 7 (completely).

Confusion

1. Since Taylor is transgender (atheist) she (he) is still figuring out who she (he) is.
2. Since Taylor is transgender (atheist), she (he) is confused about who she (he) is.
3. Since Taylor is transgender (atheist), her (his) sense of her (his) own personal identity changes more throughout their life than someone who is not transgender (atheist).
4. Because of the changes Taylor experienced in her (his) life since she (he) is transgender (atheist), it will take her (him) longer to determine who she (he) is than a non-transgender (non-atheist) person.

Deception

1. It is dishonest for Taylor to not reveal her (his) transgender (atheist) identity to others.
2. If I knew Taylor in my childhood and she (he) told me she (he) was a transgender woman (man, atheist), I would feel like I did not know her (him) at all now.
3. I felt deceived by a Taylor when she (he) came out to me as a transgender woman, (transgender man, atheist).
4. I was upset that Taylor did not tell me she (he) was Transgender (Atheist) right away.

Study 2 Questions

Prejudice

Instructions: Please respond with the answer that best reflects your opinion.

1. On the whole, Taylor is: *(1 = bad, 9 = good)*
2. On the whole, my feelings toward Taylor are: *(1 = negative, 9 = positive)*
3. On the whole, when I think about Taylor I: *(1 = I dislike her (him), 9 = I like her (him))*

Confusion and Deception

Instructions: Please indicate the extent to which you agree with each of the following statements from 1 (not at all) to 7 (completely).

Confusion

1. Taylor is still figuring out who she (he) is.
2. Taylor is confused about who she (he) is.
3. Taylor's sense of her (his) own personal identity is less stable than someone who is not transgender (atheist).
4. Taylor will take longer than a non-transgender (non-atheist) person to determine who she (he) is.

Deception

5. It is dishonest for Taylor to not reveal her (his) transgender (atheist) identity to others.
6. Learning that Taylor is transgender (atheist) made me question everything (s)he told me about herself (himself) on our date.
7. I felt deceived by a Taylor when she (he) came out to me as a transgender woman, (transgender man, atheist).
8. I was upset that Taylor did not tell me she (he) was transgender (atheist) right away.

Distrust (traits pertaining directly to distrust are italicized below)

Instructions: *Please indicate the extent to which you agree with the following statements from 1 (do not agree at all) to 7 (completely agree).*

On the whole, I think Taylor is:

1. *Trustworthy (R)*
2. Disgusting
3. Likable
4. Gross
5. Weird
6. Different
7. *Dishonest*
8. *A liar*
9. Scary
10. Kind
11. Thinking about themselves more than others
12. Thinking about others more than themselves
13. Repulsive
14. Just like everyone else
15. Unnatural

Study 1 Confusion Results

Participant Gender. As in previous research (Totton & Rios, 2021), male participants (*M* = 4.26, *SE* = .15, 95% CI = [3.87, 4.46]) perceived targets as more confusion compared to female participants (*M* = 3.35, *SE* = .13, 95% CI [3.08, 3.61]), $F(1, 244) = 16.81$, $p < .001$, $\eta^2_p = .064$.

Atheist or Transgender Condition. In support of Hypothesis 1b, the main effect of atheist or transgender condition was significant: Participants perceived greater transgender targets (*M* = 3.40, *SE* = .14, 95% *CI* [3.13, 4.67]) as more confused than atheist targets (*M* = 4.11, *SE* = .15, 95% CI [3.82, 4.40]), $F(1, 244) = 12.72$, $p < .001$, $\eta^2_p = .050$.

Intentional or Accidental Reveal Conditions. Supporting Hypothesis 2c, whether a transgender or atheist target's identity was intentionally revealed (*M* = 3.87, *SE* = .14, 95% CI [3.59, 4.15]) or was accidentally revealed (*M* = 3.87, *SE* = .14, 95% CI [3.59, 4.15]) did not significantly impact perceived confusion, $F(1, 244) = 1.46$, $p = .288$, $\eta^2_p = .006$.

Interactions. There were no significant interactions between independent variables (atheist/transgender condition by intentional/accidental reveal condition: $F(1,244) = .367$, $p = .546$, $\eta^2_p = .001$; atheist/transgender condition by participant gender: $F(1,244) = .80$, $p = .327$, $\eta^2_p = .006$).

Study 2 Confusion Results

Participant Gender. There was no significant difference between male (*M* = 3.15, *SE* = .10, 95% *CI* [2.98, 3.36]) and female (*M* = 3.01, *SE* = .11, 95% *CI* [2.80, 3.23]) participants in perceived confusion, $F(1, 358) = 1.14$, $p = .29$, $\eta^2_p = .003$.

Atheist or Transgender Condition. In support of Hypothesis 1b, the main effect of atheist or transgender condition was significant: Participants in the transgender condition (*M* = 3.51, *SE* = 0.10, 95% *CI* [3.30, 3.72]) reported greater perceived confusion than participants in the atheist condition (*M* = 2.70, *SE* = 0.11, 95% *CI* [2.48, 2.88]), $F(1, 244) = 12.72$, $p < .001$, $\eta^2_p = .083$.

Intentional or Accidental Reveal Conditions. In support of Hypothesis 2c, whether a transgender or atheist target's identity was intentionally revealed (*M* = 3.00, *SE* = .10, 95% CI [2.78, 3.19]) or was accidentally revealed (*M* = 3.20, *SE* = .10, 95% CI [3.00, 3.40]) did not significantly impact perceived confusion, $F(1, 358) = 2.10$, $p = .148$, $\eta^2_p = .006$.

Interactions. There was not a significant interaction between transgender/atheist condition and the intentional or accidental reveal condition $F(1, 358) = 0.53$, $p = .46$, $\eta^2_p = .002$. There was a significant interaction between participant gender and transgender/atheist condition on perceptions of confusion, $F(1,358) = 7.21$, $p = 0.008$, $\eta^2_p = .020$. Simple effects tests indicated that men in the trans condition (*M* = 3.79, *SE* =.14, 95% *CI* [3.51, 4.06]) reported significantly higher perceptions of confusion than men in the atheist condition (*M* = 2.56, *SE* =.14, 95% *CI* [2.29, 2.83]), $p < .001$) or women in the transgender condition (*M* = 3.24, *SE* =.16, 95% *CI* [2.93, 3.54]), $p = .009$).
